# The expansion of amino-acid repeats is not associated to adaptive evolution in mammalian genes

**DOI:** 10.1186/1471-2164-10-619

**Published:** 2009-12-18

**Authors:** Fernando Cruz, Julien Roux, Marc Robinson-Rechavi

**Affiliations:** 1 Department of Ecology and Evolution, Biophore, University of Lausanne, 1015 Lausanne, Switzerland; 2 Swiss Institute of Bioinformatics, CH-1015 Lausanne, Switzerland

## Abstract

**Background:**

The expansion of amino acid repeats is determined by a high mutation rate and can be increased or limited by selection. It has been suggested that recent expansions could be associated with the potential of adaptation to new environments. In this work, we quantify the strength of this association, as well as the contribution of potential confounding factors.

**Results:**

Mammalian positively selected genes have accumulated more recent amino acid repeats than other mammalian genes. However, we found little support for an accelerated evolutionary rate as the main driver for the expansion of amino acid repeats. The most significant predictors of amino acid repeats are gene function and GC content. There is no correlation with expression level.

**Conclusions:**

Our analyses show that amino acid repeat expansions are causally independent from protein adaptive evolution in mammalian genomes. Relaxed purifying selection or positive selection do not associate with more or more recent amino acid repeats. Their occurrence is slightly favoured by the sequence context but mainly determined by the molecular function of the gene.

## Background

Microsatellites or simple sequence repeats (SSRs) are DNA tracts composed of 1-6 bp long motifs repeated in tandem. A balance between slippage events, that increase the purity of the repeat, and point mutations, that tend to eliminate perfect repeats, determines their length distribution. However, as the slippage rate is higher than the point mutation rate, the purity of the repeated tract will be an inverse measure of the age of the SSR [1-3].

Triplet repeats are more common within coding regions [4], as they are less likely to alter the reading frame and can be translated into amino-acid repeats (AARs). AARs are frequently associated with disease [e.g. [5,6]]. Strong effects on morphology and phenotype have also been described in dog breeds [7]. Examples of AARs contributing to adaptive evolution [2,8] have been found in case studies in insects [9], plants [10,11] and mammals [12].

Genomic comparisons have shown that highly variable AARs have a higher purity in their coding sequence [13,14]. AAR expansion has been found to correlate with the non-synonymous rate of substitution [13,15,16] supporting a role of selection in their expansion. The correlation is consistent with either relaxed purifying selection, or with positive selection; the latter is suggested by case studies of adaptive evolution [9-12]. Previous studies [13,15,16] have been restricted in their taxonomic scale, did not take into account exon boundaries, and did not integrate potential confounding parameters into their analyses. Here we perform a systematic study of mammalian genomes. We contrasted AARs in positively selected genes (PSGs) and non-PSGs [17] to examine their relationship with protein adaptive evolution. We also analyzed other factors correlating with AARs in 6 high coverage mammalian genomes. The results were confirmed on a dataset of orthologous exons with wider species diversity. Thus, the relative contribution of each parameter to the expansion of AARs has been determined.

Our results indicate that AAR expansion is not causally associated to protein adaptive evolution on a genome scale. However, there is a minor contribution of the GC context surrounding the AARs for an increased slippage rate. AARs are over-represented in genes involved in DNA binding and transcriptional activity.

## Results

### Recent expansions in mammalian Positively Selected Genes

Under the hypothesis of AARs as a resource for adaptation, genes that have experienced adaptive evolution are expected to show more and more recent (i.e. purer) AARs associated with a higher substitution rate. To test this prediction, we used the PSGs identified in a thorough study of mammalian genes [17]. First, we compared the amount of repeat containing genes (RCGs) and non-repeat containing genes (non-RCGs) between positively selected genes (PSGs) and non-positively selected genes (non-PSGs) (Table 1). A Fisher's Exact Test shows a weak but significant association between repeats and positive selection (*p *= 0.042). Repeats were then split in two classes, young repeats with high purity (>= 0.9) and old repeats with low purity (<0.9) (Table 1). The PSGs have significantly more young repeats (*p *= 0.0004), suggesting that adaptive evolution in mammals could be associated with recent expansion of repeats.

**Table 1:** Counts of AARs in Positively versus non-Positively Selected Genes in Mammals

	RCGs	non-RCGs	Pure	Impure
**PSGs**	19	381	26	8
**non-PSGs**	1207	14922	2021	2448

Counts of repeat containing genes (RCGs), repeat-free genes (non-RCGs), and of number of pure and impure amino-acid repeats (AARs), of the PSGs and non-PSGs classes. These numbers were used to perform two different Fisher's Exact Tests.

We also analyzed the physical properties of the AARs. The Lehninger classification describes four categories of amino acids: acidic, basic, polar uncharged and hydrophobic amino acids [6]. All simple amino acid repeats were classified into the corresponding category for PSGs and non-PSGs (Table 2). The distribution of amino acid repeats differed significantly between PSGs and non-PSGs in a chi-square test (*p *= 0.0003). The differences remain significant after *Yate's correction for continuity *[18] (Yates' *p *= 0.001) and are mainly due to an excess of repeats of acidic and hydrophobic amino acids in the PSGs. The excess of repeats of hydrophobic AARs explains 77.3% of the differences between PSGs and non-PSGs. However this excess is essentially due to an excess of Leucine repeats. Removing these, the Chi-square is not significant after *Yate's correction for continuity *(Yates' *p *= 0.067).

**Table 2:** Physicochemical Properties of the AARs in Positively Selected versus Non-Positively Selected Mammalian Genes

	Acidic	Basic	Polar	Hydrophobic
**PSGs**	10 (0.95)	0 (-1.08)	7 (-2.51)	17 (3.23)
**non-PSGs**	970 (-0.083)	154 (0.094)	2314 (0.22)	1031 (-0.28)

Counts of amino acid categories using the Lenhinger classification for each AAR in PSGs and non-PSGs. Values shown in brackets correspond to the residuals for each cell obtained in a Pearson's χ^2 ^test.

### The correlation of amino acid repeats with positive selection and evolutionary rates is spurious

Previous studies in human and mouse have suggested that AAR expansion could be favoured by relaxed purifying selection, repeat length being associated with higher rates of non-synonymous substitutions [13,15]. While our analyses of 6 high-quality mammalian genomes confirm a positive correlation between *d*_N _and repeat length (*ρ *= 0.043, *p *= 0.002), this is very weak. A stronger correlation is observed between the average purity of AARs and *d*_N _(*ρ *= 0.111, *p *= 1.54·10^-12^), but there is a similar correlation with *d*_S _(*ρ *= 0.112, *p *= 7.8·10^-13^), and the correlation with ω, which should be most indicative of selection, is the weakest (*ρ*=0.058, *p *= 0.00017). The similar values of correlation with *d*_N _and *d*_S _may be related to the correlations between these rates (*d*_N _*vs. d*_S _*ρ *= 0.485, *p *< 2.16·10^-16^), and with the GC context surrounding the repeats (*d*_N _*vs. GC_context _ρ *= 0.115, *p *< 2.16·10^-16^; *d*_S _*vs. GC_context _ρ *= 0.478, *p *< 2.16·10^-16^). Indeed the GC_context _also correlates with the purity (*ρ *= 0.09, *p *= 4.272·10^-08^) and the number of AARs (*ρ *= 0.06, *p *< 2.16·10^-16^).

In order to disentangle the effect of these features of gene evolution we fitted the observed variation to a linear model and performed an analysis of variance [e.g. [19]]. We performed this analysis on 3 different mammalian datasets: PSGs, the 6 high-coverage genomes, and orthologous exons (Material and Methods). We detail only the analyses of the PSG dataset (Tables 3 and 4). The other two datasets, with a majority of genes under purifying selection (mean ω = 0.161 ± 0.21), provide similar results and conclusions with slight variations in the percentage of explained variance (Additional file 1, Tables S1-S4). Adaptive AAR expansions should result in high average purities (i.e., recent or frequent slippage events) and many AARs per positively selected gene. Although the contribution of evolutionary parameters is statistically significant, it is minimal and unlikely to be biologically relevant. For the average purity of the repeats on a gene, ω explains only 0.4% of the variance, while the fact of detecting adaptive evolution on any branch of the tree (i.e. significant Likelihood Ratio Test) explains <0.1% of the variance observed for the number of repeats. This shows that the enrichment for recent repeats observed using Fisher's Exact Test was a spurious association. Protein length explains 2% of the variance for AARs, which is not surprising as longer proteins have a greater potential to host repeats. Of note, it has been shown that positive selection tests are also more significant on longer proteins [e.g. [19]], which may contribute to the association between PSGs and AARs.

**Table 3:** ANOVA of Linear Model to Explain the Average Purity of the AARs in Positively Selected and Non-Positively Selected Genes

	Df	Sum Sq	Mean Sq	F value	***p-value***	Var. (%)^6^
Residuals	3616	20.5105	0.0057			97.351
GCcontext^1^	1	0.3351	0.3351	59.078	**1.94E-14**	**1.590**
Species^2^	5	0.1154	0.0231	4.0684	**0.001101**	**0.548**
ω^3^	1	0.0872	0.0872	15.3805	**8.95E-05**	**0.414**
LRT^4^	1	0.0183	0.0183	3.2305	0.072362	0.087
P. length (aa)^5^	1	0.002	0.002	0.3525	0.552754	0.009

Total	3625	21.0685	0.4714			

^1^GC content excluding the stretch containing AARs; ^2^species containing the AAR(s); ^3^omega (*d*_N_/*d*_S_) of the most significant evolutionary model; ^4^significant test for positive selection at any branch of the tree; ^5^protein length in aminoacids; ^6^proportion of variance explained.

**Table 4:** ANOVA of Linear Model to Explain the Number of AARs in Positively Selected and Non-Positively Selected Mammalian Genes

	Df	Sum Sq	Mean Sq	F value	***p-value***	Var. (%)^6^
Residuals	82096	6806.8	0.1			96.879
P. length (aa)^1^	1	168.1	168.1	2027.45	**>2.20E-16**	**2.392**
GCcontext^2^	1	48.9	48.9	590.12	**>2.20E-16**	**0.696**
LRT^3^	1	1.4	1.4	16.3141	**>3.71E-06**	**0.020**
Species^4^	5	0.8	0.2	1.9078	0.0894	0.011
ω^5^	1	0.048	0.04798	0.5787	0.4468	0.001

Total	82105	7026.01	218.748			

^1^Protein Length in aminoacids; ^2^GC content excluding the stretch containing AARs; ^3^significant test for positive selection at any branch of the tree; ^4^species containing the AAR(s); ^5^*d*_N_/*d*_S _of the most significant evolutionary model; ^6^proportion of variance explained.

The excess of leucine repeats also appears spurious, as there is no significant correlation between the ω values of each branch in the tree and the length of the leucine repeats (*ρ *= 0.36, *p *= 0.25) or their purity (*ρ *= -0.17, *p *= 0.59).

### GC rich contexts can favour the expansion of amino acid repeats

The *GC*_context _is the only parameter highly significant in both analyses of variance (on AAR purity and on AAR number). It explains only 1.6% and 0.7% of the variance, but this is 3-fold more than the percentage explained by ω or by significant evidence of positive selection. Thus GC-rich sequences appear more prone to the expansion of repeats. To explore this question, we analyzed 16 exons showing accelerated evolution in primates due to GC-biased gene conversion (gBGC) [20]. Two out of these 16 exons have AARs, or 12% of this small dataset. Interestingly, the purity of these repeats highly correlates with the GC_*context *_(ρ = 0.85, *p *= 0.002, in 10 mammalian sequences), indicating that a GC increase due to gBGC might sometimes favour the expansion of AARs.

**Figure 1:**
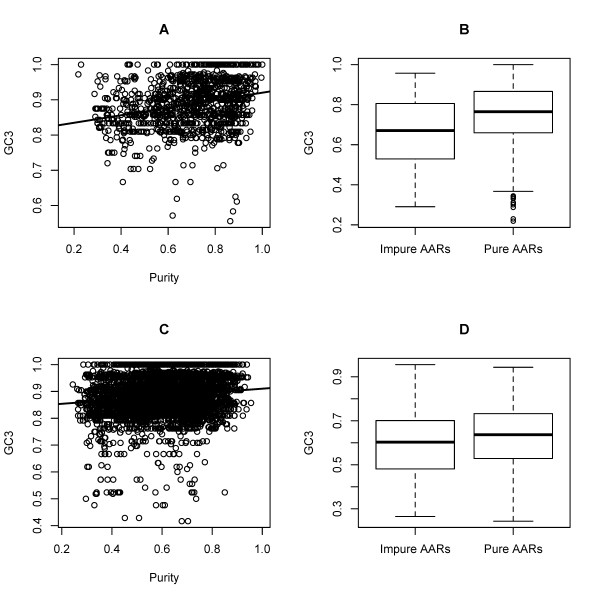
**Influence of GC content at 3^rd ^codon position on AAR purity**. GC3, GC at 3^rd ^codon positions in the sequence context of the repeats. (A) positive correlation and regression line (using least squares) between GC3 and purity in orthologous mammalian exons; (B) Average GC3 in Impure and Pure AARs in orthologous mammalian exons (*p *< 2.16·10^-16^; Welch's t-test); (C) positive correlation between GC3 and purity in mammalian genomes and regression line (using least squares); (D) Average GC3 in Impure and Pure AARs in mammalian genomes (*p *< 2.16·10^-16^; Welch's t-test).

Previous studies have also shown that nucleotide compositional constraints increasing the GC content at 3^rd ^codon positions (GC3) influence the expansion of homopolymeric AARs in mammalian and reptilian transcription factors [21]. Analyses of mammalian exons and of complete protein coding genes (Figure 1) shows that there is a weak, but highly significant, positive correlation between purity and GC3 in the DNA sequence surrounding the repeats (*ρ *= 0.28, *p *< 2.2·10^-16 ^and *ρ *= 0.126, *p *< 2.2·10^-16^, for exons and whole genes, respectively). A Welch's t-test comparing the GC3 context of exons containing pure and impure repeats indicates that genes hosting pure repeats have on average a higher GC3 than impure repeats (0.75 and 0.66 respectively, p < 2.2·10^-16^). In summary, these results consistently indicated that in mammals there is a small but significant increase of AAR expansion in regions with high GC.

### Aminoacid repeats and gene expression

The main reasons that led us to study the relationship between repeat expansion and expression levels are: 1) The observed excess of hydrophobic repeats is likely to lead to aggregation and misfolding in PSGs [22]. 2) The correlation between substitution rates and GC_*context*_, that also correlates with the average purity of AARs, has been shown to be limited by expression-related purifying selection [23]. 3) In *E. coli *it has been observed that the stability of the structure around the translation start is directly related with the expression level [24].

For the 1,057 human and 1,009 mouse genes that contain at least one AAR, we performed an analysis of variance including the expression levels in 5 representative organs as factors. The result shows that expression level has no impact on the expansion of AARs, measured as average purity or as number of repeats in the hosting gene (Additional file 1, Tables S5-S8), neither in mouse nor human.

Conversely, the number of AARs proximal to the translation start for human and mouse does not explain, in any of the 5 organs, the observed variance in the expression levels. For simplicity we show only the results obtained for the human brain (Table 5).

**Table 5:** ANOVA of a Linear Model to Explain the Expression Level of Human Genes in the Brain

	Df	Sum Sq	Mean Sq	F value	***p-value***
P. length (aa)^1^	1	2.5	2.5	0.6648	0.4151
GCcontext^2^	1	0.1	0.1	0.0178	0.894
N° AARs^3^	1	0.1	0.1	0.0226	0.8805
AARs _+30 nt_^4^	1	1	1	0.2669	0.6055
AARs _+60 nt_^5^	1	1.3	1.3	0.3386	0.5608
AARs _+90 nt_^6^	1	5.5	5.5	1.4469	0.2293
*d*_N_^7^	1	10.1	10.1	2.6413	0.1045
*Average Purity*^8^	1	0.4	0.4	0.114	0.7357
Residuals	893	3416.8	3.8		

^1^GC content excluding the stretch containing AARs; ^2^protein length in aminoacids; ^3^Number of AARs; ^4-6^Number of AARs in a window of ^4^+30 nt, ^5^+60 nt and ^6^+90 nt from translation start; ^7^Non-synomymous substitution rate; ^8^Average Purity of the AARs.

In conclusion, we can reject any simple relation between the presence of AARs or their age, and the expression level of human and mouse genes.

### Molecular function of genes hosting amino acid repeats

We studied the relation between AARs and the Gene Ontology terms (GO), for Molecular Function, Biological Process and Cell Component, of all human and mouse protein-coding genes. As very similar results were obtained for both species we will report only those obtained for human.

Genes containing AARs are enriched in a wide variety of molecular functions, mainly involved in binding, transcription and nuclear structures (Table 6); analyses accounting for purity or Biological Process of genes with AARs support these results (data not shown). Including these molecular function terms in the linear model to explain the number of AARs per gene, the total percentage of variance explained by significantly enriched GO terms is 13.9% for human and 15.2% for mouse (see Table 7 for human and Table S9 for mouse). This is not the case for average purity of AARs, for which GC context remains the main explanatory factor in human (2.73% of variance explained, Table S10). Finally, the cellular compartment nucleus is also enriched in genes with AARs, and in genes with purer AARs (GO:0005634, *p *< 6.19·10^-12^).

**Table 6:** Enrichment of Molecular Functions of Genes containing AARs

GO.ID	Term^1^	Corrected p-value^2^
GO:0050825	**ice binding**	< 1E-26
GO:0003677	**DNA binding**	4.01E-15
GO:0003700	**transcription factor activity**	1.26E-13
GO:0043565	**sequence-specific DNA binding**	5.79E-13
GO:0005199	structural constituent of cell wall	1.00E-08
GO:0004879	**ligand-dependent nuclear receptor activity**	3.15E-07
GO:0003682	**chromatin binding**	2.54E-06
GO:0003723	RNA binding	7.63E-05
GO:0008270	zinc ion binding	0.000303826
GO:0004969	histamine receptor activity	0.0008013
GO:0045735	nutrient reservoir activity	0.0008013
GO:0003702	RNA polymerase II transcription factor activity	0.001116964
GO:0003676	nucleic acid binding	0.001580342
GO:0003705	RNA polymerase II transcription factor activity, enhancer binding	0.009862154
GO:0003735	structural constituent of ribosome	0.02671
GO:0005249	voltage-gated potassium channel activity	0.049858667
GO:0004386	helicase activity	0.065105625
GO:0016563	transcription activator activity	0.13355
GO:0003714	transcription corepressor activity	0.13355
GO:0005179	hormone activity	0.199622105

^1 ^In bold terms overrepresented also for genes hosting the highest average purity of their AARs; ^2 ^FDR < 20%.

**Table 7:** Percentage of Explained Variance of the Number of Aminoacid Repeats

Factor	Pr(>F)	Var. (%)
***ice binding***	<2.20E-16	5.869336006
P. length	<2.20E-16	2.718369933
*structural constituent of cell wall*	<2.20E-16	1.965991088
***DNA binding***	<2.20E-16	1.544242393
GC context	<2.20E-16	0.754548334
*structural constituent of ribosome*	<2.20E-16	0.597911216
***Transcription factor activity***	<2.20E-16	0.575348528
*hormone activity*	<2.20E-16	0.554521432
*histamine receptor activity*	<2.20E-16	0.553219739
*nucleic acid binding*	<2.20E-16	0.547145169
*Voltage-gated potassium channel activity*	<2.20E-16	0.488135064
***ligand-dependent nuclear receptor activity***	2.33E-12	0.348853859
***sequence-specific DNA binding***	3.01E-09	0.249491255
*RNA binding*	1.70E-07	0.193952332
*d*_S_	1.25E-06	0.166616768
***chromatin binding***	3.29E-06	0.153165936
*RNA polymerase II transcription factor activity, enhancer binding*	3.63E-06	0.151864242
*d*_N_	6.19E-06	0.144921877
*nutrient reservoir activity*	0.0004664	0.086779567
*transcription corepressor activity*	0.0054142	0.054671127
ω	0.0134962	0.043389783
RNA polymerase II transcription factor activity	0.0240022	0.03601352
helicase activity	0.1667501	0.013450833
zinc ion binding	0.198911	0.011715242
transcription activator activity	0.4614908	0.003905081

In italics GO Terms that remain significant after Bonferroni Correction. In Bold functions enriched in pure AARs.

The ice binding molecular function (GO:0050825) is overrepresented. But this excess disappears after excluding the Alanine repeats. This appears to be an annotation bias, as genes containing alanine-rich repeats are attributed this function by partial sequence similarity with the InterPro entry IPR000104 (Antifreeze protein, type I), a special glycoprotein identified in marine teleosts from polar oceans[25].

## Discussion

In mammals, a positive correlation between *d*_N _and repeat length is weak but statistically significant. This result is congruent with previous analyses in smaller datasets of human and mouse genomes [13,15]. The purity of the AARs per gene or exon shows a similar trend. But these weak correlations can be explained by the influence of the GC context surrounding the repeat. High GC content can generate a sequence context more prone to slippage[21,26-28] and thus expansion of AARs. Indeed we found an example of this in exons that have experienced GC-biased gene conversion in primates. Similarly, while there is an increase in the amount of recent AARs in mammalian PSGs, these recent expansions are better explained by GC content than by positive selection acting on codons. Therefore it seems that, in contradiction to previous reports [15], the expansion of AARs is not causally associated with substitution rates. While purifying selection limits the expansion of AARs[e.g. [29]], this appears to be distinct from the selective pressure on individual (aligned) amino acid sites. That means that these repeats are experiencing not only different mutational processes, but also particular selective constraints, leading to a more complex scenario of evolution.

Our analyses, even of individual exons, suggest that increased substitution rates are not usually linked to the presence of AARs. However, it is possible that in some particular cases, as has been suggested for *Drosophila*, the expansion of AARs can produce compensatory changes on the neighbouring sites to accommodate the perturbation generated by the repeat[30]. We also cannot exclude the existence of adaptive evolution related with AARs[7,8], in the absence of a good reference neutral model for tri-nucleotide expansions in proteins. But our results do show that the selective pressure as measured by codon models is not related with putative adaptive evolution of AARs.

AARs in mammalian genes do not seem to affect gene expression significantly. Unlike repeats which disrupt the reading frame, and have a strong effect on replication and transcription stability[31], the tri-nucleotide repeats might be constrained in a different way. It seems that repeats located in the promoter region[32] have a stronger influence on transcription than do AARs, even those near the transcription start.

The analyses of molecular function confirmed an enrichment in the transcription factor, DNA binding, molecular transducers and binding categories that is consistent with previous studies of polymorphic repeats [26,33,34]. The overrepresentation of transcription factor categories supports the existence of *trans *effects, as these repeats might alter the expression of the target genes and end up producing dramatic changes on the phenotype[7]. However, while the ice-binding protein is involved in hypothermic resistance in some antartic fishes vertebrates[25,35], its overrepresentation in alanine-rich mammalian genes is probably due to an annotation bias.

In general, we found that AARs are located in proteins that interact with DNA, RNA, ligands or other proteins, so it is likely that they contribute to adapt or modulate the interaction capacity of these proteins. Longer proteins and repeat-rich proteins tend to have a higher connectedness within interaction networks, suggesting that they contribute to an enlarged interaction surface and constitute more flexible subunits[36]. Some AAR have been recently associated to the presence of repeats to specific domains, such as signal peptides or transmembrane regions[16], pointing to their role in facilitating molecular interactions of extreme importance. For example, in the Drosophila ARC 70 cofactor complex, the -130 and -230 subunits contain an expansion of glutamine residues, a prevalent feature of sequence-specific activators in *Drosophila*[37].

## Conclusions

Despite the appealing idea of an adaptive role of the expansion of amino acid repeats, we can rule out a link with adaptive evolution in mammalian protein-coding genes as measured by codon models. Genome-wide, GC content is more relevant to amino acid repeat expansions than substitution rates. Amino acid repeats are under strong functional constraints and expand preferentially in transcription factors and nuclear genes involved in DNA and/or protein interactions. Why some genes accumulate more and most recent amino acid repeats requires further study in a network context, to shed light on the evolutionary dynamics and function of these mutations.

## Methods

### Positively Selected Genes (PSGs)

A recent study in mammals[17] performed a thorough analysis for detecting positive selection in six mammalian genomes. A likelihood ratio test for positive selection on any branch of the phylogeny reported 400 Positively Selected Genes (PSGs), and 16,129 genes that have not experienced any detected positive selection in mammals (non-PSGs). Alignments for these genes were downloaded from the author's website http://compgen.bscb.cornell.edu/projects/mammal-psg/lrtall.txt and screened for repeats.

### High-quality Mammalian Genomes

To study the relationship of multiple factors that could be influencing the expansion of repeats in mammalian genomes, we used mammalian assemblies with high coverage (ranging from 6-11×) and their corresponding Ensembl 50 Genes[38]. We compared the genomes of 2 primates (*Homo sapiens *NCBI36 and *Pan troglodytes *CHIMP2.1), 2 rodents (*Mus musculus *NCBIM37 and *Rattus norvegicus *RGSC3.4) and 2 domestic species (*Bos taurus *Btau_3.1 and *Canis familiaris *Canfam 2.0).

For each mammalian genome, we downloaded all the known protein coding genes, with exception of dog and chimp genomes where, in order to gather the largest accurate dataset, we used the "known by projection" set. The repeat analyses are restricted to non-redundant one-to-one orthologues to an equidistant outgroup, dog in the case of rodents and primates, and human for the domestic species. We filtered the genes by keeping the protein corresponding to the longest transcript and excluding all coding sequences that did not begin with a start codon. Finally the number of genes that were screened for repeats in each species was 13,926 human, 11,120 chimpanzee, 13,921 mouse, 10,360 rat, 7,073 cow and 7,834 dog genes.

### Orthologous Exons

We downloaded 1,168 orthologous exons alignments including 9 to12 mammalian species, from the OrthoMam database [39]. This is a curated database that contains the amino acid and coding sequence alignments for each particular exon. The inclusion of these alignments allowed studying local AAR expansions without biases due to regional differences in substitution rates and GC context along the whole gene. The exon trees were built using PHYML (substitution model = JTT, estimated proportion of invariable sites, four categories, estimated gamma, initial tree with BIONJ) [40]. Evolutionary rates for each branch where obtained running the *free-ratios *model in PAML 4.1 [41] and keeping *d*_N_, *d*_S _and ω convergent values of 5 replicate runs. Non-convergent or 999 values were not considered in further analyses.

### Homo-polymeric Amino-acid Repeats and Purity

As in many previous studies we focused on perfect homo-polymeric amino-acid repeats, where we assume that the expansion of a tri-nucleotide by slippage gave birth to the repetition of a single amino-acid motif within the protein. To consider that an amino-acid repeat appeared by polymerase slippage a minimum threshold of 5 units was frequently used in the literature [e.g. [8,26]]. We used a minimum number of 7 units. The reasons for this are, first, to increase the significance level[6] and, second, to increase the chance that a repeat locus shows length polymorphism [42,43].

The *purity *of the nucleotide sequence coding the amino-acid repeat was calculated following the method described by Laidlaw et al. in 2007[8]that is summarized in the equation below;(1)

where *m *is the total number of nucleotides coding the amino-acid repeat and *n *the number of interruptions or nucleotide changes with respect to the canonical codon (the most frequent or most likely to have experienced expansion by slippage). The presence of AARs was considered for each species independently of the presence of that repeat in orthologues.

### Summary of parameters and estimates

Each gene was screened for homo-polymeric amino acid repeats within the corresponding protein sequence. The following parameters were calculated:

*i) Weighted Average Purity of the Repeats of a Gene*: the weighted average of the *purity *estimates of every amino-acid repeat in the protein sequence of a gene. The weighting is based on the length of the coding sequence of each individual repeat, as described in the following equation;(2)

where *n *is the total number of AARs on the protein-coding gene, *l *is the length in bp of each individual repeat, *P *is the corresponding Purity and *L *the sum in bp of the length of all AARs in the gene. This measure allowed us to compare if certain genes contain purer AARs than others. Note that the vast majority of the cases correspond to genes hosting only one AAR. (Additional file 2, Figures SF1 and SF2)

*ii) d*_N _and *d*_S_: sitewise maximum likelihood estimates of *d*_N _and *d*_S _for each orthologous pair were downloaded from Ensembl [38].

*iii) GC context *(%GC): the GC content of gene after excluding all regions encoding repeats[44]. Similarly, we estimated GC3 as the GC content on third codon positions of the full repeat-free coding dna of the gene or exon. These parameters depict the sequence context in which repeats are born.

### Gene Expression data

Microarray data of mouse and human tissues were downloaded from ArrayExpress (E-AFMX-4 and E-AFMX-5)[45]. E-AFMX-4 uses an Affymetrix Custom Array - Novartis Mouse (A-AFFY-39) and E-AFMX-5 uses an Affymetrix Custom Array - Novartis Human (A-AFFY-40). Mapping of Ensembl gene on Affymetrix probesets from these chips was taken from http://biogps.gnf.org/downloads/. E-AFMX-5 also uses an Affymetrix GeneChip Human Genome HG-U133A (A-AFFY-33), whose mapping to Ensembl genes was downloaded from BioMart [46].

We extracted expression data for 5 organs in mouse (cerebral cortex, liver, kidney, testis and heart) and human (brain, liver, kidney, testis and heart). Raw CEL files were renormalized using the package gcRMA [47] of Bioconductor version 2.2[48]. We used the "affinities" model of gcRMA, which uses mismatch probes as negative control probes to estimate the non-specific binding of probe sequences. The normalized values of expression are in log2 scale, which attenuates the effect of outliers. Expression values were averaged between replicates and between multiple probes mapped to a same gene. Probes mapping to more than one gene were discarded.

### GO term enrichment

Over and under representation of GO terms [49] was tested by means of a Fisher exact test, using the Bioconductor package topGO version 1.8.1 [50]. The reference set was all Ensembl genes used in the repeats analysis. The GO annotation of Ensembl genes was downloaded from BioMart. The "elim" algorithm of topGO was used, allowing to decorrelate the graph structure of the gene ontology, reducing non-independence problems. Gene ontology categories with a FDR < 20% were reported.

## Authors' contributions

FC designed the project, gathered the genomic data, performed most of the evolutionary and statistical analyses, and wrote the original manuscript. JR gathered the expression and gene ontology data, and performed the analysis of GO Term enrichment. MRR supervised the work, provided critical comments about the statistical analyses and the biological discussion, and revised thoroughly the manuscript. All authors have read and approved the final manuscript.

## Supplementary Material

Additional file 1 **Supplementay Tables**. A PDF file containing additional Tables S1-S11. These tables contain analyses of variance with factors sorted by percentage of explained variance. Further details are provided as footnotes accompanying each table. Click here for file

Additional file 2 A PDF file containing additional Figures SF1-SF5. Further details are provided as footnotes accompanying each Figure. Click here for file
