# Supplementary material for: The expansion of amino-acid repeats is not associated to adaptive evolution in mammalian genes
Source: BMC Genomics. 2009 Dec 18;10:619. doi: 10.1186/1471-2164-10-619 (PMC2806350; doi:10.1186/1471-2164-10-619)
Supplement: Additional file 1 — Supplementay Tables. A PDF file containing additional Tables S1-S11. These tables contain analyses of variance with factors sorted by percentage of explained variance. Further details are provided as footnotes accompanying each table. [file 1471-2164-10-619-S1.PDF]

# Supplementary Tables

**Table S1 – Explained Variance of average purity in 6 mammalian genomes**

| | Df | Sum Sq. | Mean Sq. | F-value | Pr(>F) | Signif. | Expl. Var. (%) |
|---|---|---|---|---|---|---|---|
| **Residuals** | 4000 | 18,7857 | 0,0047 | | | | 97,10931563 |
| **Species** | 5 | 0,2815 | 0,0563 | 11,9892 | 1,53E-11 | *** | **1,455163893** |
| **GCcontext** | 1 | 0,1731 | 0,1731 | 36,8493 | 1,40E-09 | *** | **0,894809485** |
| ω | 1 | 0,0527 | 0,0527 | 11,215 | 0,0008189 | *** | **0,272423223** |
| **dN** | 1 | 0,0482 | 0,0482 | 10,2667 | 0,0013652 | ** | **0,249161278** |
| **Protein Length** | 1 | 0,0035 | 0,0035 | 0,7405 | 0,3895639 | | 0,018092624 |
| **dS** | 1 | 0,0002 | 0,0002 | 0,0359 | 0,8496383 | | 0,001033864 |
| **TOTAL** | 4010 | 19,3449 | 0,3387 | | | | |

**Table S2 – Explained Variance of the number of AARs in 6 mammalian genomes**

| | Df | Sum Sq. | Mean Sq. | F value | Pr(>F) | Signif. | Expl. Var. (%) |
|---|---|---|---|---|---|---|---|
| **Residuals** | 64223 | 7938 | 0,1 | | | | 96,69638942 |
| **Protein Length** | 1 | 196,3 | 196,3 | 1587,958 | <2,20E-16 | *** | **2,391219607** |
| **Species** | 5 | 33,5 | 6,7 | 54,234 | <2,20E-16 | *** | **0,408078741** |
| **GCcontext** | 1 | 32,5 | 32,5 | 263,066 | <2,20E-16 | *** | **0,395897286** |
| **dS** | 1 | 5,3 | 5,3 | 43,13 | 5,16E-11 | *** | **0,064561711** |
| **ω** | 1 | 1,9 | 1,9 | 15,308 | 9,14E-05 | *** | **0,023144764** |
| **dN** | 1 | 1,7 | 1,7 | 13,405 | 0,0002511 | *** | **0,020708473** |
| **TOTAL** | 64233 | 8209,2 | 244,5 | | | | |

**Table S3 – Explained Variance of average purity in orthologous mammalian exons**

| | Df | Sum Sq | Mean Sq | F value | Pr(>F) | Signif. | Expl. Var. (%) |
|---|---|---|---|---|---|---|---|
| **Residuals** | 1024 | 4,333 | 0,0042 | | | | 90,12250671 |
| **Species** | 24 | 0,2066 | 0,0086 | 2,0344 | 0,002379 | ** | **4,297094366** |
| **Protein Length** | 1 | 0,148 | 0,148 | 34,9853 | 4,52E-09 | *** | **3,078267019** |
| **GCcontext** | 1 | 0,0785 | 0,0785 | 18,5577 | 1,81E-05 | *** | **1,632729466** |
| **dS** | 1 | 0,0158 | 0,0158 | 3,7447 | 0,053252 | . | 0,328625803 |
| **Corrected ω**[1] | 1 | 0,0136 | 0,0136 | 3,2118 | 0,073403 | . | 0,28286778 |
| **dN** | 1 | 0,008 | 0,008 | 1,8813 | 0,170489 | | 0,166392812 |
| **ω** | 1 | 0,0044 | 0,0044 | 1,0374 | 0,308661 | | 0,091516047 |
| **TOTAL** | 1054 | 4,8079 | 0,2811 | | | | |

[1] Calculated as $(d_N+1)/(d_S+1)$, allows calculating the omega ratio when dS=0.

**Table S4 – Explained Variance of the number of AARs in orthologous mammalian genomes**

| | Df | Sum Sq | Mean Sq | F value | Pr(>F) | Signif. | Expl. Var. (%) |
|---|---|---|---|---|---|---|---|
| **Residuals** | 10487 | 1716,34 | 0,16 | | | | 97,97411849 |
| **Species** | 24 | 11,82 | 0,49 | 3,0089 | 1,07E-06 | *** | **0,674723004** |
| **GCcontext** | 1 | 9,95 | 9,95 | 60,8102 | 6,88E-15 | *** | **0,567977486** |
| **Protein Length** | 1 | 4,59 | 4,59 | 28,0322 | 1,22E-07 | *** | **0,262011725** |
| **dN** | 1 | 3,97 | 3,97 | 24,269 | 8,51E-07 | *** | **0,226620163** |
| **ω** | 1 | 3,47 | 3,47 | 21,2051 | 4,18E-06 | *** | **0,198078581** |
| **Corrected ω**[1] | 1 | 1,63 | 1,63 | 9,9855 | 0,001582 | ** | **0,093045558** |
| **dS** | 1 | 0,06 | 0,06 | 0,3756 | 0,540005 | | 0,00342499 |
| **TOTAL** | 10517 | 1751,83 | 24,32 | | | | |

[1] Calculated as $(d_N+1)/(d_S+1)$, allows calculating the omega ratio when dS=0.

**Table S5 – Explained Variance of the average purity in human with expression levels in 5 organs as additional factors**

| | Df | Sum Sq | Mean Sq | F value | Pr(>F) | Signif. | Exp. Var. (%) |
|---|---|---|---|---|---|---|---|
| Residuals | 894 | 4,2576 | 0,0048 | | | | 96,71967288 |
| **GC context** | 1 | 0,1343 | 0,1343 | 28,2014 | 1,38E-07 | *** | **3,050885961** |
| heart | 1 | 0,0039 | 0,0039 | 0,8234 | 0,3644 | | 0,088596093 |
| testis | 1 | 0,0025 | 0,0025 | 0,5325 | 0,4658 | | 0,056792367 |
| kidney | 1 | 0,0018 | 0,0018 | 0,3705 | 0,5429 | | 0,040890504 |
| brain | 1 | 0,001 | 0,001 | 0,2092 | 0,6475 | | 0,022716947 |
| liver | 1 | 0,0005 | 0,0005 | 0,1015 | 0,7501 | | 0,011358473 |
| dN | 1 | 0,0004 | 0,0004 | 0,0829 | 0,7734 | | 0,009086779 |
| Total | 901 | 4,402 | 0,1492 | | | | |

**Table S6 – Explained Variance of the number of AARs in human with expression levels in 5 organs as additional factors**

| | Df | Sum Sq | Mean Sq | F value | Pr(>F) | Signif. | Exp. Var. (%) |
|---|---|---|---|---|---|---|---|
| Residuals | 11673 | 2109,11 | 0,18 | | | | 99,34573716 |
| **GC context** | 1 | 12,51 | 12,51 | 69,2451 | <2E-16 | *** | **0,58926048** |
| liver | 1 | 1,01 | 1,01 | 5,5867 | 0,01811 | * | **0,047574187** |
| dN | 1 | 0,15 | 0,15 | 0,8163 | 0,3663 | | 0,007065473 |
| kidney | 1 | 0,09 | 0,09 | 0,5183 | 0,47156 | | 0,004239284 |
| heart | 1 | 0,08 | 0,08 | 0,4264 | 0,5138 | | 0,003768252 |
| brain | 1 | 0,03 | 0,03 | 0,1444 | 0,70397 | | 0,001413095 |
| testis | 1 | 0,02 | 0,02 | 0,1028 | 0,7485 | | 0,000942063 |
| Total | 11680 | 2123 | 14,07 | | | | |

**Table S7 – Explained Variance of the average purity in mouse with expression levels in 5 organs as additional factors**

| | Df | Sum Sq | Mean Sq | F value | Pr(>F) | Signif. | Exp. Var. (%) |
|---|---|---|---|---|---|---|---|
| Residuals | 894 | 4,2576 | 0,0048 | | | | 99,37679434 |
| liver | 1 | 0,01541 | 0,01541 | 3,6889 | 0,05519 | . | 0,359685363 |
| kidney | 1 | 0,00687 | 0,00687 | 1,6434 | 0,20029 | | 0,160352916 |
| dN | 1 | 0,00156 | 0,00156 | 0,3735 | 0,54128 | | 0,036412016 |
| testis | 1 | 0,0013 | 0,0013 | 0,3112 | 0,57714 | | 0,030343347 |
| **GC context** | 1 | 0,00128 | 0,00128 | 0,3064 | 0,5801 | | **0,029876526** |
| heart | 1 | 0,00027 | 0,00027 | 0,0657 | 0,79775 | | 0,00630208 |
| cortex | 1 | 0,00001 | 0,00001 | 0,0028 | 0,95781 | | 0,00023341 |
| Total | 901 | 4,2843 | 0,0315 | | | | |

**Table S8 – Explained Variance of the number of AARs in mouse with expression levels in 5 organs as additional factors**

| | **Df** | **Sum Sq** | **Mean Sq** | **F value** | **Pr(>F)** | **Signif.** | **Exp. Var. (%)** |
|---|---|---|---|---|---|---|---|
| Residuals | 10107 | 1476,68 | 0,15 | | | | 99,60137327 |
| **GC context** | 1 | 3,05 | 3,05 | 20,9051 | 4,88E-06 | *** | **0,205721069** |
| kidney | 1 | 1,36 | 1,36 | 9,3082 | 0,002287 | ** | **0,091731362** |
| liver | 1 | 0,74 | 0,74 | 5,0713 | 0,024347 | * | **0,049912653** |
| heart | 1 | 0,4 | 0,4 | 2,7531 | 0,097096 | . | 0,026979812 |
| cortex | 1 | 0,21 | 0,21 | 1,4078 | 0,235444 | | 0,014164401 |
| dN | 1 | 0,09 | 0,09 | 0,615 | 0,432938 | | 0,006070458 |
| testis | 1 | 0,06 | 0,06 | 0,3997 | 0,527282 | | 0,004046972 |
| Total | 10114 | 1482,59 | 6,06 | | | | |

**Table S9 – Explained Variance of the number of AARs in mouse with Molecular Functions' GO Terms as additional factors**

| | Pr(>F) | Signif. | Expl. Var. (%)[a] |
|---|---|---|---|
| *ice binding* | <2,20E-16 | *** | 6,2414 |
| P. length | <2,20E-16 | *** | 2,94 |
| *structural constituent of cell wall* | <2,20E-16 | *** | 2,3188 |
| *DNA binding* | <2,20E-16 | *** | 1,9856 |
| *structural molecule activity* | <2,20E-16 | *** | 1,1645 |
| *nucleic acid binding* | <2,20E-16 | *** | 0,7887 |
| *histamine receptor activity* | <2,20E-16 | *** | 0,6462 |
| *ligand dependent nuclear receptor activity* | <2,20E-16 | *** | 0,5253 |
| GC context | 5,94E-16 | *** | 0,4630 |
| *structural constituent of ribosome* | 4,82E-15 | *** | 0,4334 |
| *profilin binding* | 7,74E-11 | *** | 0,2989 |
| *transcription factor activity* | 1,49E-09 | *** | 0,2582 |
| dS | 3,45E-08 | *** | 0,2151 |
| *voltage gated potassium channel activity* | 4,51E-06 | *** | 0,1485 |
| *chromatin binding* | 2,22E-05 | *** | 0,1270 |
| *p53 binding* | 0,0003049 | *** | 0,0918 |
| *sequence specific DNA binding* | 0,0004869 | *** | 0,0857 |
| *transcription factor binding* | 0,0038186 | ** | 0,0590 |
| transcription corepressor activity | 0,0174995 | * | 0,0398 |
| *zinc ion binding* | 0,0432591 | * | 0,0285 |
| $\omega$ | 0,0565458 | . | 0,026 |
| transcription repressor activity | 0,0610786 | . | 0,0248 |
| RNA binding | 0,1459721 | | 0,0149 |
| dN | 0,187283 | | 0,0122 |
| transcription activator activity | 0,2670875 | | 0,0089 |
| double stranded DNA binding | 0,275614 | | 0,0084 |
| nutrient reservoir activity | 0,641019 | | 0,0014 |
| Residuals | | | |

**[a]the total percentage of variance explained by significant molecular functions is 15.17%**

**Table S10 – Explained Variance of the purity of AARs in human with Molecular Functions' GO Terms as additional factors**

| | Pr(>F) | Signif. | Expl. Var. (%) |
|---|---|---|---|
| GC context | 2,33E-07 | *** | 2,730557125 |
| P. length | 0,5339 | | - |
| ω | 0,9593 | | - |
| dN | 0,2352 | | - |
| dS | 0,8552 | | - |
| nucleic acid binding | 0,2735 | | - |
| DNA binding | 0,1926 | | - |
| chromatin binding | 0,7544 | | - |
| transcription factor activity | 0,6198 | | - |
| RNA polymerase II transcription factor activity | 0,2354 | | - |
| RNA polymerase II transcription factor activity enhancer binding | 0,7475 | | - |
| transcription corepressor activity | 0,3521 | | - |
| RNA binding | 0,1445 | | - |
| structural constituent of ribosome | 0,111 | | - |
| helicase activity | 0,9786 | | - |
| ligand.dependent nuclear receptor activity | 0,4922 | | - |
| histamine receptor activity | 0,1647 | | - |
| hormone activity | 0,0782 | . | - |
| structural constituent of cell wall | 0,2884 | | - |
| voltage gated potassium channel activity | 0,641 | | - |
| zinc ion binding | 0,7783 | | - |
| transcription activator activity | 0,3968 | | - |
| sequence specific DNA binding | 0,221 | | - |
| nutrient reservoir activity | 0,2098 | | - |
| ice binding | 0,6347 | | - |
| Residuals | | | |
| Total | | | |

**Table S11 – Explained Variance of purity in 435 Poly-Q repeats belonging 6 mammalian genomes.**

| | Pr(>F) | Signif. | Var. (%)[a] |
|---|---|---|---|
| Residuals | | | 88.67198739 |
| Species | 3.6053 | **0.0033266** | 3.787445199 |
| Prot. Length (aa) | 14.9435 | **0.0001282** | 3.139803513 |
| GC3 | 13.0694 | **0.0003364** | 2.74618383 |
| $d_N$ | 5.6855 | **0.0175464** | 1.194589966 |
| GC1 | 1.529 | 0.2169479 | 0.321532357 |
| $\omega$ | 0.6183 | 0.4321133 | 0.130443732 |
| $d_S$ | 0.0359 | 0.8498293 | 0.008009703 |
| GC2 | 2.05E-05 | 0.9963899 | 4.31E-06 |

**[a]the total percentage of variance explained by the lineal model**

Variance explained by the GC3 is 2.3-fold the explained by $d_N$. The latter is less significant. The selective pressure measured as $\omega = d_N/d_S$ is not significant.
