# Supplementary material for: The expansion of amino-acid repeats is not associated to adaptive evolution in mammalian genes
Source: BMC Genomics. 2009 Dec 18;10:619. doi: 10.1186/1471-2164-10-619 (PMC2806350; doi:10.1186/1471-2164-10-619)
Supplement: Additional file 2 — A PDF file containing additional Figures SF1-SF5. Further details are provided as footnotes accompanying each Figure. [file 1471-2164-10-619-S2.PDF]

Supplementary Figures

Figure SF1

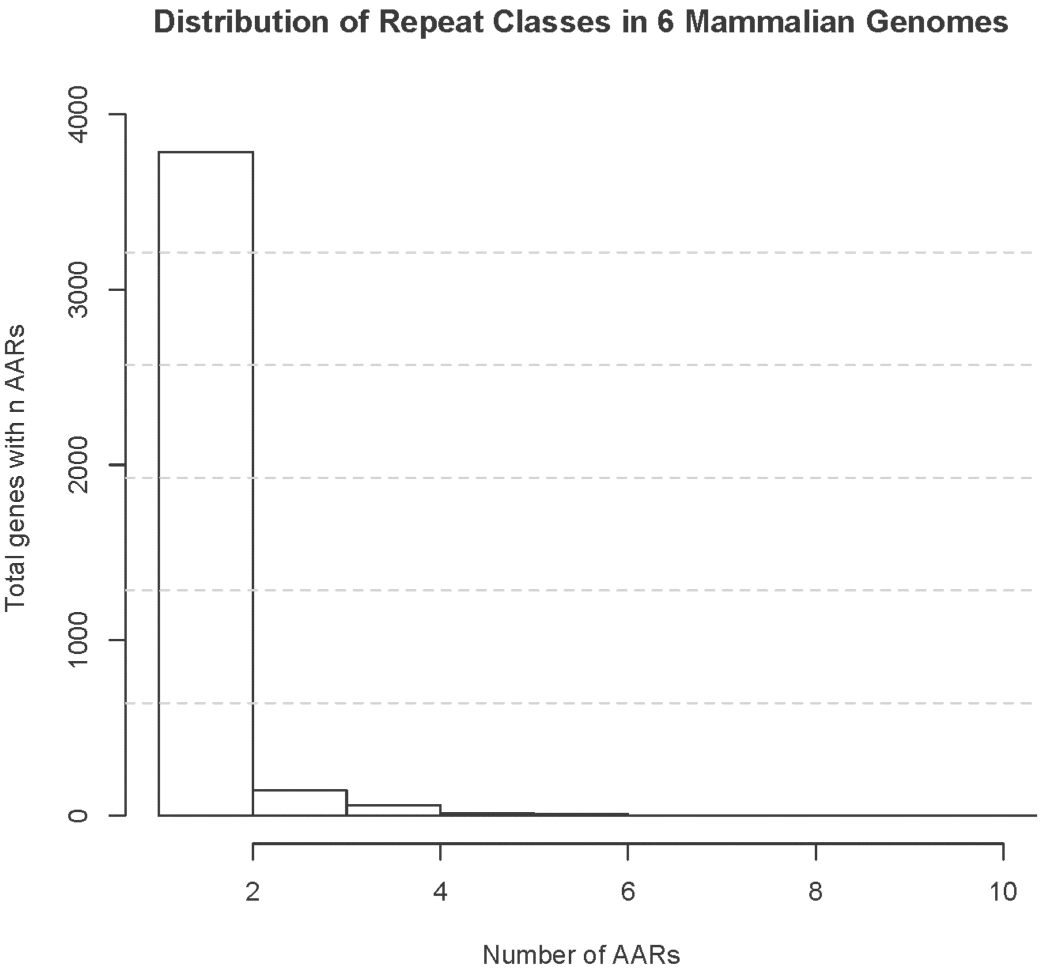

Distribution of different gene classes with varying number of AARs in the 6 mammalian genomes from Ensembl (~700-900 genes per genome). The grey dash lines indicating 1%-5% of the total number of protein-coding genes.

Figure SF2

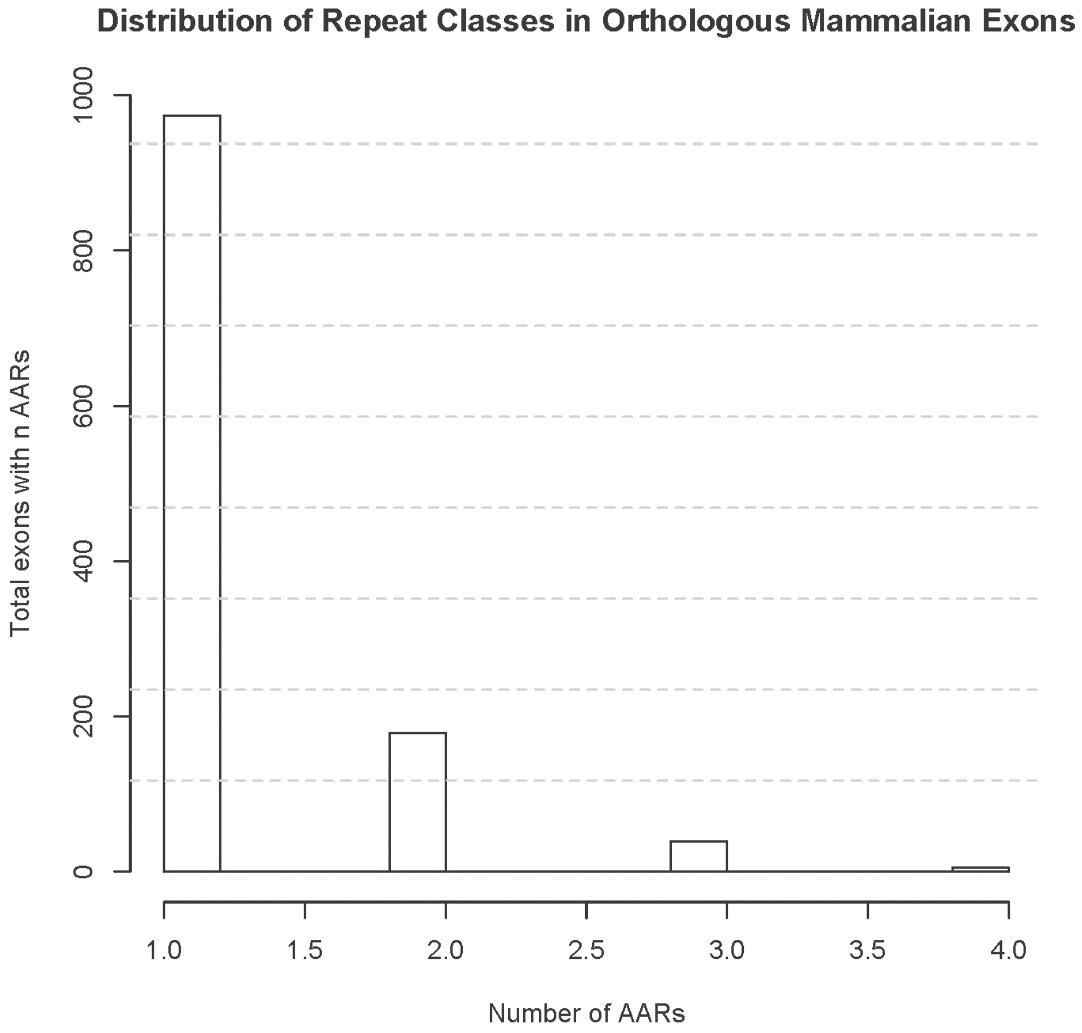

Distribution of different exon classes with varying number of AARs in the OrthoMam exons. The grey dash lines indicating 1% to 8% of the total number of exon sequences.

Figure SF3 –Distribution of AARs based on the number of intrerruptions of the repeated tract (Mammalian Genomes)

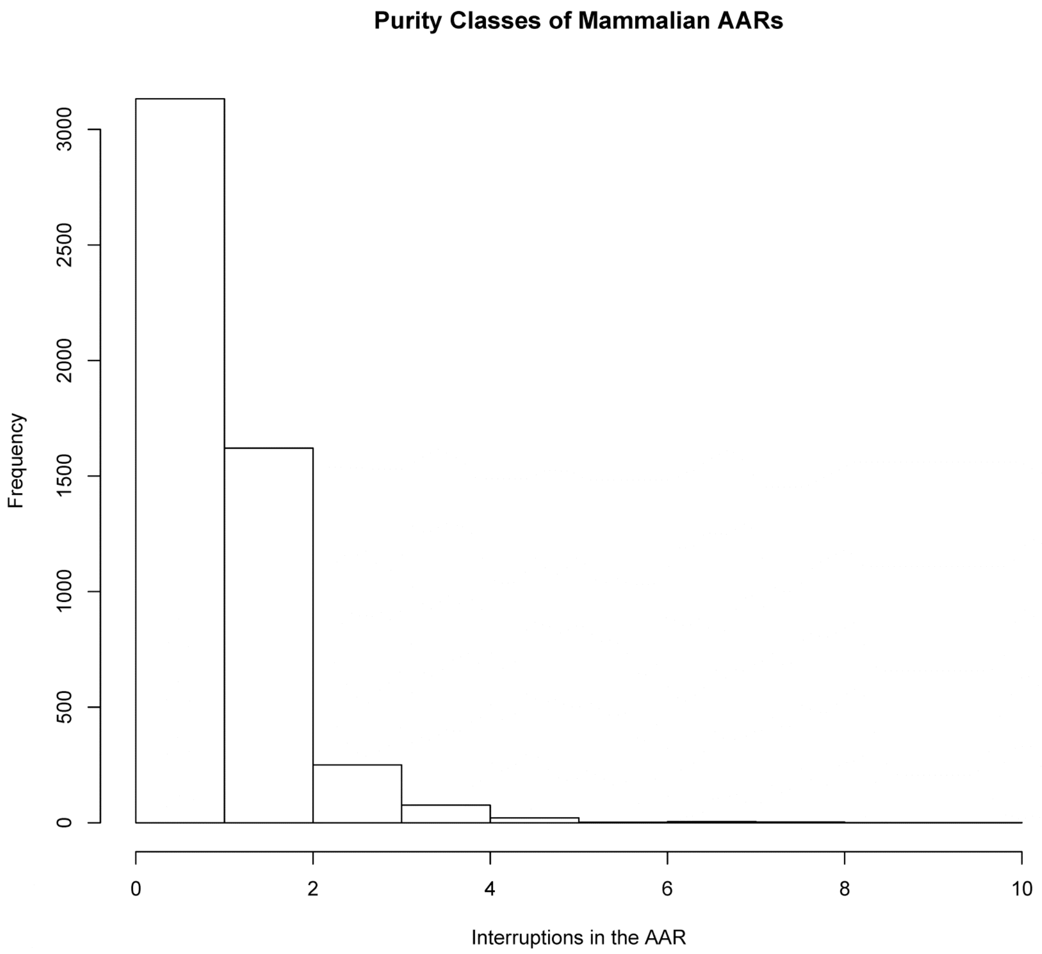

Represents the distribution of AAR classes with decreasing purity. AARs with 0 interruptions in their coding sequence will have a purity of 1. 1 interruption in a 7 units AAR (21bp length) will correspond to a purity of 0.95. Above 1 interruptions or point mutation we observe a steady decline in number of loci (rarely polymorphic or prone to slippage AARs), and the number of AAR almost disappear with 4 or more interruptions (inactive slippage)

Figure SF4 - Relationship among GC3 and Purity in Mammalian Poly-Q

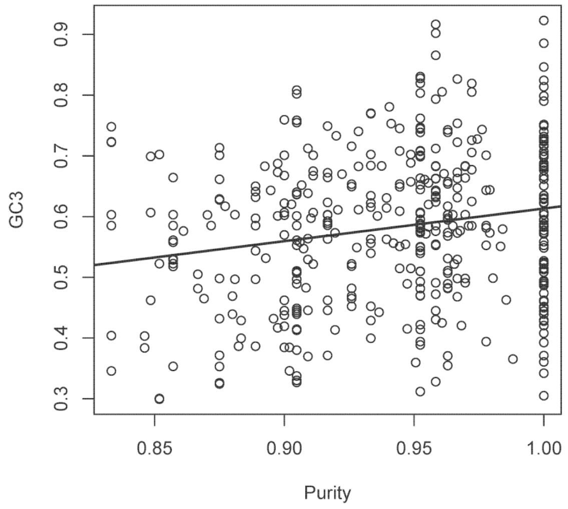

Positive correlation and regression line (using least squares) between GC3 and purity in poly-glutamine mammal repeats ($\rho = 0.174$, $p < 0.0002671$)

Figure SF5 - Relationship among d_N and Purity in Mammalian Poly-Q

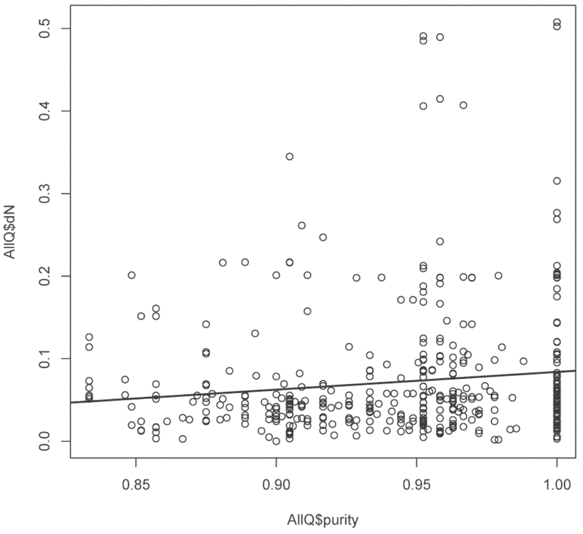

Positive correlation and regression line (using least squares) between d_N and purity in poly-glutamine mammal repeats ($\rho = 0.14$, $p < 0.0042$)
